# Retraction: Unpacking the optimistic mindset of business students towards entrepreneurship

**DOI:** 10.1371/journal.pone.0342209

**Published:** 2026-02-05

**Authors:** The PLOS One Editors

Following publication of this [1] article, the following concerns were raised:

- The article may not adhere to the journal’s third and fourth publication criteria [2]. Specifically:
  - The study sample is not appropriate for the research question and is insufficiently defined. Additionally, the representativeness and generalizability of the sample is unknown,
  - Key descriptive statistics including means, standard deviations, and correlations as well as sampling details and control variables are not reported.
- [1] cites an article ( [3]; reference 13 in [1]) that was retracted prior to the publication of [1] due to concerns about manipulation of the publication process.

PLOS consulted a member of the *PLOS One* Editorial Board who advised that the data obtained from undergraduate students in entrepreneurship courses rather than working entrepreneurs cannot support the conclusions regarding entrepreneurship, the role of networks, and the other treatment variables. They also stated that the reporting of the study population and statistical analyses are insufficient.

The response received from the corresponding author, AJK, did not resolve the concerns.

The *PLOS One* Editors retract this article due to the above unresolved concerns about the reliability of the reported research and reliance on retracted work cited in Reference 13, as well as compliance with PLOS policies on Authorship and Ethical Publishing Practice. We regret that the issues were not identified prior to the article’s publication.

AJK did not agree with the retraction. HM, SF, WUH, and HU either did not respond directly or could not be reached.
